# Association of the IgG *N*-glycome with the course of kidney function in type 2 diabetes

**DOI:** 10.1136/bmjdrc-2019-001026

**Published:** 2020-04-28

**Authors:** Sunny S Singh, Ralph Heijmans, Claudia K E Meulen, Aloysius G Lieverse, Olga Gornik, Eric J G Sijbrands, Gordan Lauc, Mandy van Hoek

**Affiliations:** 1 Internal Medicine, Erasmus MC, Rotterdam, Zuid-Holland, Netherlands; 2 Internal Medicine, Maxima Medical Centre, Eindhoven, Noord-Brabant, Netherlands; 3 Faculty of Pharmacy and Biochemistry, University of Zagreb, Zagreb, Croatia

**Keywords:** IgG N-glycans, diabetes type 2, N-glycosylation, kidney function, nephropathy

## Abstract

**Introduction:**

Inflammatory processes are thought to be involved in kidney function decline in individuals with type 2 diabetes. Glycosylation of immunoglobulin G (IgG) is an important post-translation process affecting the inflammatory potential of IgG. We investigated the prospective relationship between IgG *N*-glycosylation patterns and kidney function in type 2 diabetes.

**Research design and methods:**

In the DiaGene study, an all-lines-of-care case–control study (n=1886) with mean prospective follow-up of 7.0 years, the association between 58 IgG *N*-glycan profiles and estimated glomerular filtration rate (eGFR) and albumin-to-creatinine ratio (ACR) per year and during total follow-up was analyzed. Models were adjusted for clinical variables and multiple comparisons.

**Results:**

Eleven traits were significantly associated with eGFR change per year. Bisecting GlcNAc in fucosylated and fucosylated disialylated structures and monosialylation of fucosylated digalactosylated structures were associated with a faster decrease of eGFR. Fucosylation of neutral and monogalactosylated structures was associated with less eGFR decline per year. No significant associations between IgG glycans and ACR were found.

**Conclusions:**

In type 2 diabetes, we found IgG *N-*glycosylation patterns associated with a faster decline of kidney function, reflecting a pro-inflammatory state of IgG. eGFR, but not ACR, was associated with IgG glycans, which suggests these associations may represent renal macroangiopathy rather than microvascular disease.

**Significance of this study**

**What is already known about this subject?**

- Inflammatory processes play a role in chronic kidney disease in type 2 diabetes.
- The variation in glycan sugar residues attached to the conserved glycosylation sites of the immunoglobulin G (IgG) Fc part influences IgG effector function, modulating the immune response from either pro-inflammatory to an anti-inflammatory response or vice versa.
- The link between IgG glycosylation and renal function in type 2 diabetes has never been investigated.

**What are the new findings?**

- We found pro-inflammatory IgG *N*-glycosylation patterns associated with a faster decline of kidney function estimated glomerular filtration rate, but not albumin-to-creatinine ratio, possibly representing renal macroangiopathy.

**How might these results change the focus of research or clinical practice?**

- Our findings suggest the involvement of the immune system in the pathophysiology of diabetic nephropathy in type 2 diabetes, representing a novel target for future biomarker and therapeutics developments.

## Introduction

Chronic kidney disease (CKD) is one of the most common complications in type 2 diabetes mellitus, despite extensive preventive efforts. Apart from known risk factors, a large residual risk remains for developing CKD,^1 2^ which may be partly explained by processes such as inflammation. Biomarkers that provide information, in addition to known risk factors, can aid in better prediction and tailored treatment to prevent and delay kidney function decline.^3 4^

**Figure 1:**
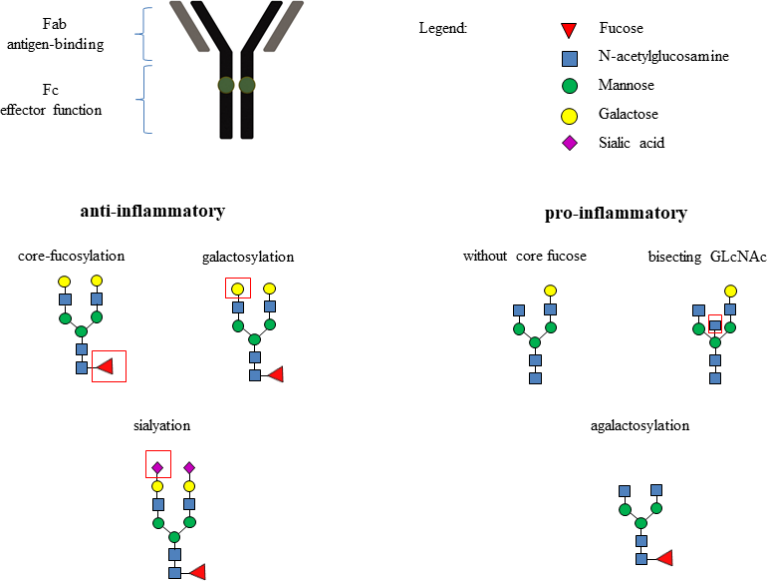
Functional implications and inflammatory associations of IgG glycosylation. Dark-green spots on IgG molecule are conserved glycosylation sites Asn297 to which biantennary glycans are attached.

A growing body of literature recognizes the association between type 2 diabetes and the *N*-linked glycosylation of proteins.^5^ *N*-Linked glycosylation is a co-translational and post-translational modification of proteins, influencing their function.^6 7^ *N*-Glycans affect the stability, activity and targeting of proteins, as well as cell–cell and host–pathogen interaction.^6 7^ These complex oligosaccharides are assembled by the coordinated action of a range of glycosyltransferases and glycosidase enzymes, and are attached to the nitrogen (N) atom of asparagine side chains of proteins within a specific sequon.^6^ *N*-Glycosylation patterns of the IgG *N*-glycome and total plasma *N*-glycome have been associated with estimated glomerular filtration rate (eGFR) in non-diabetic individuals and those with type 1 diabetes.^8 9^ Furthermore, a cross-sectional study showed that characteristic patterns of the total plasma *N*-glycome are associated with renal function in type 2 diabetes.^10^ However, the link between the immunoglobulin G (IgG) *N*-glycome and renal function in type 2 diabetes patients has never been investigated. Immunoglobulin G (IgG) is the most abundant antibody in the human body, involved in infectious and inflammatory processes through several mechanisms: antigen neutralization, promotion of phagocytosis, microbial killing via opsonization and macrophage activation, complement activation and induction of ADCC (antibody-dependent cellular cytotoxicity).^11^ IgG consist of a fragment antigen binding (Fab) domain and fragment crystallizable (Fc) domain, which interacts with Fc gamma receptors (FcyR). Single biantennary glycans are attached to each heavy chain on the Fc part asparagine-297 (Asn297). They are essential for binding to the FcyRs. The receptor interaction is lost if no glycans are attached. The variation in glycan sugar residues attached influences IgG effector function, modulating the immune response from either pro-inflammatory to an anti-inflammatory response or vice versa. The variations consist of the addition of bisecting *N*-acetylglucosamine (GlcNac), fucose to core, as well as galactose and sialic acid to the arms of the biantennary glycan. Bisecting GlcNac and afucosylated *N*-glycans have a pro-inflammatory effect, while the addition of galactose and sialic acid has an anti-inflammatory effect on IgG (figure 1).^12^ IgG glycosylation patterns are highly variable between individuals, but show good temporal stability in a single healthy individual.^13^ Yet, IgG patterns are known to change in a single individual because of alterations in a person’s health status.^13–15^ IgG glycosylation contains a genetic, heritable component, as well an environmental component. Because of these features, glycosylation is considered an interface between genetic background and environment.^14 16^,^12 17 18^ Because type 2 diabetes and chronic kidney disease are multifactorial diseases displaying features of chronic inflammation, IgG *N*-glycosylation is promising as a biomarker, but also from a pathophysiological perspective.^19^

We hypothesized that specific IgG-glycan profiles prospectively associate with kidney function in type 2 diabetes, and could be a potential biomarker in the future We, therefore, investigated the prospective relationship between IgG *N*-glycosylation and the course of kidney function during follow-up in type 2 diabetes individuals.

## Research design and methods

### Study design

Data were derived from the DiaGene study, and the study characteristics have previously been described in more detail.^20^ Briefly, the DiaGene study is a large multicenter prospective cohort study with 1886 patients with type 2 diabetes with prospective follow-up on kidney function (mean follow-up time=7.0 years). Data were collected from two hospitals in and around the city of Eindhoven, the Netherlands. In addition, the local primary care diagnostic center participated in the study. All participants provided informed written consent.

### Definitions

Type 2 diabetes was defined as fasting plasma glucose ≥7.0 mmol/L and/or a non-fasting plasma glucose level ≥11.1 mmol/L measured at least at two separate time points, treatment with oral glucose-lowering medication or insulin, and/or the diagnosis of type 2 diabetes as registered by a medical specialist. Microalbuminuria was defined as urinary albumin-to-creatinine ratio (ACR) ≥2.5 for men or ≥3.5 for women. Macroalbuminuria was defined as ACR ≥12.5 for men or ≥17.5 for women. Around the moment of inclusion, laboratory data were obtained, which that is, contained glycated hemoglobin (HbA1c), total cholesterol, high-density lipoprotein (HDL) cholesterol and urinary ACR. The eGFR was calculated with the Modification of Diet in Renal Disease formula and mean arterial pressure (MAP) was defined as (systolic blood pressure+2×diastolic blood pressure)/3. Non-HDL was calculated by subtracting HDL from total cholesterol. To calculate eGFR and ACR percentage change during total follow-up, the value at baseline and the last known value during follow-up were used. The eGFR and ACR percentage change per year were determined by dividing the percentage difference during total follow-up by total follow-up time in years.

### IgG *N*-glycome analysis

Plasma for IgG *N*-glycosylation analysis was available in 1837 cases, and 22 samples failed quality control, resulting in 1815 cases. Isolation, release and labeling of IgG glycans has been described in detail previously.^5 14^ In total, 24 IgG glycan peaks were measured by Waters Acquity UPLC instrument. All chromatograms were separated into 24 peaks and the amount of glycans in each peak was expressed as percentage of total integrated area. From these direct traits, an additional 34 derived IgG glycan traits were calculated based on their structural similarities. As a result, characteristics of the 24 direct glycan peaks are reflected in the derived traits. A detailed description can be found in online supplementary table S1. The distribution of the IgG glycan peaks was analyzed by visual inspection of QQ plots and showed no major deviations from normality. IgG glycan expressions were globally normalized and log transformed, and all measurements were adjusted for batch effects by applying ComBat (R-package sva). Before statistical analyses were performed, all IgG glycan traits were centered and scaled to have mean 0 and SD 1.

10.1136/bmjdrc-2019-001026.supp1 Supplementary data

### Glycan nomenclature

A glycan structure formula is described as follows: FA_x_BG_x_S_x_. The abbreviations stand for F=fucose, A=antennae, B=bisection, G=galactose and S=sialic acid, and x indicates the number of the particular feature. When, for example, F or S is not present in the structure formula, the glycan has no fucose or sialic acids attached. For some glycans, for example, GP9 with structure formula FA2[3]G1, [3] means the antennae (A2) are bound on the third carbon bond of mannose of the glycan core.

### Statistical analysis

Mean and SD of cohort characteristics were determined. Linear regression models were used to investigate associations between the 58 IgG glycan patterns and eGFR and ACR. Four dependent variables were constructed: percentage change of eGFR during total follow-up, percentage change ACR during total follow-up, eGFR percentage change per year and ACR percentage change per year. The basic model for each dependent variable included age and sex and their interaction. Different full models were constructed, for adjusting for confounders. These full models always contained smoking, MAP, body mass index (BMI), HbA1c, duration of type 2 diabetes, non-HDL and HDL. In addition, in the full models of dependent variables eGFR and ACR percentage change during total follow-up, we adjusted for duration of follow-up. Furthermore, we additionally adjusted the full models of the ACR analyses for ACE-inhibitor use. We had 80% power to detect Cohen’s effect size f^2^: 0.009 based on 1815 cases and 11 predictors.^21^ Correction for multiple comparisons was performed by the Benjamini-Hochberg method.^22^ Statistical analyses were carried out using IBM SPSS Statistics V.25.0.

## Results

### Cohort characteristics

Characteristics of the study population are shown in table 1. All participants were aged between 27 and 94 with a mean of 65.2 years; 53.6% were male, mean BMI was 30.5 kg/m^2^, mean HbA1c was 7.0% and mean duration of type 2 diabetes at inclusion was 10.1 years. On average, the eGFR and ACR percentage change per year were −2.3% and 162.2%, respectively. Microalbuminuria was present in 2.8% of all patients at baseline and in 5.5% at end of follow-up. Macroalbuminuria was present in 0.2% of the patients at both instances.

**Table 1:** Characteristics of the study population

Characteristic	Cases (n=1886)
Age (years)	65.2 (±10.6)
Sex (% male)	53.6
BMI (kg/m^2^)	30.5 (±5.4)
HDL cholesterol (mmol/L)	1.17 (±0.32)
Non-HDL cholesterol (mmol/L)	3.12 (±0.90)
Never smoked (%)	23.3
Former smoker (%)	50.7
Current smoker (%)	16.2
MAP (mm Hg)	98.9 (±10.8)
HbA1c (%)	7.0 (±1.1)
HbA1c (mmol/L)	53.31 (11.58)
Duration type 2 diabetes at inclusion (years)	10.1 (±8.4)
Duration of follow-up (years)	6.9 (±2.1)
eGFR change during total follow-up (%)	−7.7 (±24.5)
eGFR change per year (%)	−2.3 (±24.9)
ACR change during total follow-up (%)	800.5 (±3626.8)
ACR change per year (%)	162.2 (±678.5)
Normoalbuminuria (ACR <30 mg/g) (%)	90.5
Microalbuminuria (ACR 30–300 mg/g) (%)	2.8
Macroalbuminuria (ACR >300 mg/g) (%)	0.2
Normoalbuminuria at follow-up (%)	79.2
Microalbuminuria at follow-up (%)	5.5
Macroalbuminuria at follow-up (%)	0.2

Unless stated otherwise, mean (±SD) are given.

ACR, albumin-to-creatinine ratio; BMI, body mass index; eGFR, estimated glomerular filtration rate; HDL, high-density lipoprotein; MAP, mean arterial pressure.

### IgG glycan associations with eGFR percentage change per year

In table 2, significant associations between IgG *N*-glycans and eGFR percentage change per year are shown for both models. Results for all 58 investigated IgG glycan peaks and derived traits are shown in online supplementary table S2.

**Table 2:** Statistically significant associations of IgG glycan traits with eGFR change per year

Glycan traits	Basic model		Full model	
	**β**	**P value**	**β**	**P value**
GP4 (FA2)	−1.13	6.17E−03	−0.13	NS
GP6 (FA2B)	−1.38	7.76E−04	−0.76	6.18E−03
GP8 (A2BG1; FA2[6]G1)	1.18	2.28E−03	0.61	NS
GP9 (FA2[3]G1)	1.05	5.65E−03	0.44	NS
GP14 (FA2G2)	1.12	1.01E−02	0.49	NS
Derived traits				
FG2S1/(FG2+FG2S1+FG2S2)	−0.27	NS	−0.74	2.66E−03
FBStotal/FStotal	−1.16	3.94E−03	−0.31	NS
FBS2/FS2	−1.38	4.16E−04	−0.78	3.20E−03
FBS2/(FS2+FBS2)	−1.21	2.25E−03	−0.74	5.89E−03
G0n	−1.32	1.84E−03	−0.39	NS
G1n	1.43	3.51E−04	0.34	NS
Fn	0.71	NS	0.70	5.70E−03
FG1n/G1n	0.80	NS	0.71	4.71E−03
FG2n/G2n	1.08	6.09E−03	0.51	NS
FBn	−0.73	NS	−0.72	4.73E−03
FBG1n/G1n	−0.77	NS	−0.70	5.24E−03
FBG2n/G2n	−1.27	1.42E−03	−0.75	4.11E−03
FBn/Fn	−0.77	NS	−0.78	2.20E−03
FBn/Fntotal	−0.74	NS	−0.74	3.89E−03

Basic model: age, sex and their interaction.

Full model: smoking, mean arterial pressure, body mass index, HbA1c, duration of type 2 diabetes, non-HDL, HDL.

A p value was considered statistically significant for the basic model when p<1.01E−02 and for the full model when p<6.18E−03. Regression coefficient (beta) and p value per association, with FDRα used as cut-off for significance.

B, bisection; eGFR, estimated glomerular filtration rate; F, fucose; G, galactose; HDL, high-density lipoprotein; N, neutral; NS, non-significant; S, sialylation.

In the full model, 1 IgG *N-*glycan peak and 10 derived traits were significantly associated with eGFR percentage change per year. Agalactosylated fucosylated biantennary glycans with bisecting GlcNAc (GP6; FA2B) were associated with a faster decrease of eGFR. In addition, bisecting GlcNAc in fucosylated disialylated structures (FBS2/FS2 and FBS2/(FS2+FBS2)), fucosylated structures with bisecting GlcNAc (FBn, FBG1n/G1n, FBG2n/G2n, FBn/Fn and FBn/Fntotal) and the percentage of monosialylation of all fucosylated digalactosylated structures without bisecting GlcNAc (FG2S1/(FG2+FG2S1+FG2S2)) were also associated with a faster decrease of eGFR.

Traits associated with less eGFR decline per year were fucosylated structures without bisecting GlcNAc in neutral (Fn) and monogalactosylated structures (FG1n/G1n).

### IgG glycan associations with eGFR percentage change during total follow-up

Associations between all 58 investigated IgG glycan patterns and the eGFR percentage change during total follow-up are shown in online supplementary table S3.

In the basic model, five IgG *N-*glycan peaks and three derived traits were significantly associated with eGFR percentage change during total follow-up. However, after the adjustment for clinical risk factors, no associations remained significant. In the basic model, patterns associated with a larger decrease in eGFR over time were fucosylated biantennary glycans with (GP6: FA2B) and without bisecting GlcNAc (GP4: FA2) and agalactosylated structures (G0n). Monogalactosylated (GP8: A2BG1, FA2[6]G1; GP9: FA2[3]G1), digalactosylated (GP14: FA2G2), core fucosylated biantennary glycans without bisecting GlcNAc and monogalactosylated (G1n) and digalactosylated structures (G2n) were associated with a smaller decrease in eGFR over time.

### IgG glycan associations with ACR

In both models, we did not find any significant association between IgG glycan patterns and both ACR percentage change and ACR percentage change per year. Associations between all 58 investigated IgG glycan patterns and the ACR percentage change during total follow-up and per year are shown in online supplementary table S4 and S5, respectively.

## Discussion

In the present study, we describe the prospective association of IgG *N-*glycome with the prospective course of kidney function in type 2 diabetes. After adjustment for confounders, monosialylation, bisecting GlcNAc and fucosylation with bisecting GlcNAc were associated with a faster decrease of eGFR per year. Bisecting GlcNAc and fucosylated bisected IgG reflect biological aging as well as a pro-inflammatory state of IgG. In contrast, fucosylation without bisecting GlcNAc was associated with less eGFR decline per year. These non-bisected fucosylated IgG can implicate an anti-inflammatory IgG effector function. No significant associations were found between IgG glycan patterns and ACR.

Although the pathogenesis of nephropathy in diabetes is only partly understood, it is known that hyperglycemia leads to activation of inflammatory pathways, which attributes to the risk of vascular complications due to tissue injury.^23 24^ More specifically, in diabetic nephropathy, high blood glucose levels cause renal cells to release humoral mediators, pro-inflammatory cytokines and growth factors.^25 26^ Subsequently, GFR deteriorates due to changes in kidney structure and function, such as thickening of the glomerular basement membrane and glomerulosclerosis.^25–27^ Thus, inflammation plays a major role in the development of diabetic nephropathy. Changes in IgG glycan composition can modify the effector function of IgG, hereby contributing to inflammation.^28^ The role of IgG glycans in diabetic nephropathy is supported by an in vivo study, in which Fcγ receptor–deficient mice developed less renal hypertrophy, inflammation and fibrosis.^29^ IgG *N*-glycans change the Fcy receptor affinity, hereby regulating inflammatory processes such as antibody-dependent cellular phagocytosis and ADCC. IgG glycan modulation has also been shown to attenuate the development of ANCA-mediated glomerulonephritis.^30^ IgG *N-*glycosylation patterns have already been associated with kidney function in non-diabetic individuals and type 1 diabetes.^8 9^ Key properties of IgG glycosylation are galactosylation, sialylation, fucosylation and bisecting GlcNAc. We will discuss our findings and associated literature accordingly.

Galactosylation of IgG *N-*glycans is essential for the initiation of the anti-inflammatory signaling cascade through the inhibitory receptor FcγRIIB.^31^ In addition, the pro-inflammatory activity of the complement component C5a can be inhibited by highly galactosylated immune complexes.^28 32 33^ Therefore, galactosylation reflects an anti-inflammatory state of IgG. A lack of galactosylation is known to activate the complement system via the lectin pathway by binding to mannose-binding lectin and via the alternative pathway,^28 32 34–36^ which induces inflammation. In our study, agalactosylated structures were associated with a faster decline of kidney function, whereas monogalactosylated structures and GP14 (FA2G2) were associated with less kidney function decline per year in the basic model. When adjusting for clinical covariates, such as HbA1c, these associations did not remain significant. However, in the extensive model, GP6 (FA2B), of which the absence of galactose is an important feature, was significantly associated with a faster decline of kidney function independent of clinical covariates. These findings are in line with other studies, as agalactosylated IgG glycan structures were also associated with a more rapid eGFR decline in type 1 diabetes and a higher risk of CKD in the non-diabetic population.^8 9^

In previous studies, the sialylation of IgG glycans decreased the affinity of Fcγ receptors, leading to anti-inflammatory activity^18 37^: the addition of sialic acid alters IgG from a pro-inflammatory to an anti-inflammatory state. In our study, GP6 (FA2B), characterized by the absence of sialic acid, was associated with faster decrease in kidney function. In line with our finding, the percentage of sialylated structures in IgG glycans was also decreased in non-diabetic patients with CKD.^9^ In our study, a faster decrease in kidney function was also associated with monosialylation of fucosylated digalactosylated structures without bisecting GlcNAc, which we cannot completely explain. It could be a result of a relative increase, as a consequence of a (non-significant) reduction in disialylated structures of the same type. This is supported by the overall negative direction of association of the percentage of sialylation in these structures.

High abundance of IgG core fucose serves as a “safety switch”, as it decreases the affinity for the FcγRIIIA and FcγRIIIB receptors.^38–40^ As a result, core fucose prevents ADCC.^38–40^ However, the presence of bisecting GlcNac in fucosylated structures has the opposite effect, as it increases the affinity for the receptors mentioned above.^41 42^ The combination of fucose with bisecting GlcNac is associated with a pro-inflammatory state of IgG. In our study, fucosylated structures with bisecting GlcNAc were associated with a faster decrease of kidney function after adjustment for clinical variables. This was also reflected in the association with GP6 (FA2B), which entails the same feature. Moreover, fucosylated structures without bisecting GlcNAc were associated with less annual kidney function decline. Similar results were found in a non-diabetic population, as sialylated core fucosylated glycans with bisecting GlcNac were associated with lower eGFR.^8^ Interestingly, monosialylation with presence of bisecting GlcNac was associated with lower eGFR in the non-diabetic population,^8^ whereas in our study, disialylation in combination with bisecting GlcNAc was associated with a faster decrease of eGFR.

In our study, we did not find an association of IgG glycosylation with prospective changes in albuminuria. Increase of ACR is one of the first clinical signs of diabetic nephropathy. However, studies in both types of diabetes have also shown that higher albuminuria levels do not necessarily result into renal function decline.^43–45^ A portion of diabetic patients indeed presents with normoalbuminuria in combination with a decline in renal function.^46 47^ It has been shown that these patients tend to have a higher age and a more advanced CKD with lower eGFR and lower hemoglobin.^48^ This suggests that, for these patients, the kidney function decline may be related to macrovascular rather than to microvascular disease, as may also be the case in our population of older patients with type 2 diabetes. As another study^45^ has shown that intrarenal vascular disease is dependent of eGFR, but independent of ACR, the associations between IgG glycans and renal function found in our study may indicate renal macroangiopathy rather than microvascular glomerulopathy. Associations between IgG glycans and ACR were described in type 1 diabetes.^9^ The large portion of both microalbuminuria and macroalbuminuria at baseline in this type 1 diabetes population (13.45% and 10.27% vs 2.8% and 0.2% in our study, respectively) may explain this difference.

Strengths of our study are the use of a large and unselected cohort of patients with type 2 diabetes from all lines of care with prospective follow-up. A variety of clinical features was available to include in our analysis. Furthermore, this is the first study addressing the IgG *N*-glycome in relation to kidney disease in type 2 diabetes and prospectively. Although we have performed our study with great care, some limitations remain. First, IgG glycans were measured at baseline. As a result, we have not investigated temporal sequence glycan composition changes, which could have influenced the kidney function during follow-up. However, IgG glycans are known to be fairly stable unless a major physiological change takes place.^13^ Moreover, for biomarker purposes, a marker that predicts well with one measurement, rather than sequential measurements would be preferred. Second, residual confounding due to yet unknown factors affecting both the IgG *N-*glycome and kidney function cannot be excluded. However, by adjusting for many clinical factors, we have reduced this to a minimum. Third, our study has only evaluated the IgG *N-*glycome, which reflects glycans originating from IgG. Glycans present on other plasma proteins, as can be measured in the total *N-*glycome, may also associate with the prognosis of kidney function.

To conclude, we found IgG *N-*glycosylation patterns, reflecting a pro-inflammatory state of IgG at baseline, associated prospectively with a faster decline of kidney function in patients with type 2 diabetes. eGFR, but not ACR, was associated with IgG glycans, which suggests these associations may represent renal macroangiopathy rather than microvascular disease.

Our findings suggest the involvement of the immune system in the pathophysiology of diabetic nephropathy in type 2 diabetes, representing a novel target for future biomarker and therapeutics development. Mendelian randomization studies and in-depth in vitro studies could provide more insight into the causality of the discovered relationships as well as the pathophysiological mechanisms.
