# Supplementary material for: Association of the IgG N-glycome with the course of kidney function in type 2 diabetes
Source: BMJ Open Diabetes Res Care. 2020 Apr 28;8(1):e001026. doi: 10.1136/bmjdrc-2019-001026 (PMC7213753; doi:10.1136/bmjdrc-2019-001026)
Supplement: Supplementary data [file bmjdrc-2019-001026supp001.pdf]

Supplementary Tables

Supplementary Table S1 - Derived traits description

Description of direct glycan peaks and derived traits measured in this study(5). Structure abbreviations are as follows: F, α-1,6-linked core fucose; A, number of antenna's; B, bisecting GlcNAc β1-4 linked to β1-3 mannose; M, number of mannose residues; Gx, number of β1-4 linked galactoses; [3]G1, galactose on the antenna of the α1-3 linked mannose; [6]G1, galactose on the antenna of the α1-6 linked mannose; Sx, number of sialic acids linked to galactose.

GROUP	DESCRIPTION			FORMULA
Total IgG glycans (neutral + charged)	GP1	FA1	*The percentage of FA1 glycan in total IgG glycans*	*GP1 / GP * 100*
	GP2	A2	*The percentage of A2 glycan in total IgG glycans*	*GP2 / GP * 100*
	GP3	A2B	*The percentage of A2B glycan in total IgG glycans*	*GP3 / GP * 100*
	GP4	FA2	*The percentage of FA2 glycan in total IgG glycans*	*GP4 / GP * 100*
	GP5	M5	*The percentage of M5 glycan in total IgG glycans*	*GP5 / GP * 100*
	GP6	FA2B	*The percentage of FA2B glycan in total IgG glycans*	*GP6 / GP * 100*
	GP7	A2[3]G1	*The percentage of A2G1 glycan in total IgG glycans*	*GP7 / GP * 100*
	GP8	A2BG1<br>FA2[6]G1	*The percentage of FA2[6]G1 glycan in total IgG glycans*	*GP8 / GP * 100*
	GP9	FA2[3]G1	*The percentage of FA2[3]G1 glycan in total IgG glycans*	*GP9 / GP * 100*
	GP10	FA2[6]BG1	*The percentage of FA2[6]BG1 glycan in total IgG glycans*	*GP10 / GP * 100*
	GP11	FA2[3]BG1	*The percentage of FA2[3]BG1 glycan in total IgG glycans*	*GP11 / GP * 100*
	GP12	A2G2	*The percentage of A2G2 glycan in total IgG glycans*	*GP12 / GP * 100*
	GP13	A2BG2	*The percentage of A2BG2 glycan in total IgG glycans*	*GP13 / GP * 100*
	GP14	FA2G2	*The percentage of FA2G2 glycan in total IgG glycans*	*GP14 / GP * 100*
	GP15	FA2BG2	*The percentage of FA2BG2 glycan in total IgG glycans*	*GP15 / GP * 100*
	GP16	FA2[6]G1S1<br>FA2[3]G1S1	*The percentage of FA2G1S1 glycan in total IgG glycans*	*GP16 / GP * 100*
	GP17	A2G2S1	*The percentage of A2G2S1 glycan in total IgG glycans*	*GP17/ GP * 100*
	GP18	A2BG2S1<br>FA2G2S1	*The percentage of FA2G2S1 glycan in total IgG glycans*	*GP18 / GP * 100*
	GP19	FA2BG2S1	*The percentage of FA2BG2S1 glycan in total IgG glycans*	*GP19 / GP * 100*
	GP20*	FA2FG2S1	*The percentage of FA2FG2S1 in total IgG glycans**	*GP20 / GP * 100*
	GP21	A2G2S2	*The percentage of A2G2S2 glycan in total IgG glycans*	*GP21 / GP * 100*

	GP22	A2BG2S2	*The percentage of A2BG2S2 glycan in total IgG glycans*	*GP22 / GP * 100*
	GP23	FA2G2S2	*The percentage of FA2G2S2 glycan in total IgG glycans*	*GP23 / GP * 100*
	GP24	FA2BG2S2	*The percentage of FA2BG2S2 glycan in total IgG glycans*	*GP24 / GP * 100*

Total IgG glycans - derived parameters	FGS/(FG+FGS)	*The percentage of sialylation of fucosylated galactosylated structures without bisecting GlcNAc in total IgG glycans*	*SUM(GP16 + GP18 + GP23) / SUM(GP16 + GP18 + GP23 + GP8 + GP9 + GP14) * 100*
	FBGS/(FBG+FBGS)	*The percentage of sialylation of fucosylated galactosylated structures with bisecting GlcNAc in total IgG glycans*	*SUM(GP19 + GP24) / SUM(GP19 + GP24 + GP10 + GP11 + GP15) * 100*
	FGS/(F+FG+FGS)	*The percentage of sialylation of all fucosylated structures without bisecting GlcNAc in total IgG glycans*	*SUM(GP16 + GP18 + GP23) / SUM(GP16 + GP18 + GP23 + GP4 + GP8 + GP9 + GP14) * 100*
	FBGS/(FB+FBG+FBGS)	*The percentage of sialylation of all fucosylated structures with bisecting GlcNAc in total IgG glycans*	*SUM(GP19 + GP24) / SUM(GP19 + GP24 + GP6 + GP10 + GP11 + GP15) * 100*
	FG1S1/(FG1+FG1S1)	*The percentage of monosialylation of fucosylated monogalactosylated structures without bisecting GlcNAc in total IgG glycans*	*GP16 / SUM(GP16 + GP8 + GP9) * 100*
	FG2S1/(FG2+FG2S1+FG2S2)	*The percentage of monosialylation of fucosylated digalactosylated structures without bisecting GlcNAc in total IgG glycans*	*GP18 / SUM(GP18 + GP14 + GP23) * 100*
	FG2S2/(FG2+FG2S1+FG2S2)	*The percentage of disialylation of fucosylated digalactosylated structures without bisecting GlcNAc in total IgG glycans*	*GP23 / SUM(GP23 + GP14 + GP18) * 100*
	FBG2S1/(FBG2+FBG2S1+FBG2S2)	*The percentage of monosialylation of fucosylated digalactosylated structures with bisecting GlcNAc in total IgG glycans*	*GP19 / SUM(GP19 + GP15 + GP24) * 100*
	FBG2S2/(FBG2+FBG2S1+FBG2S2)	*The percentage of disialylation of fucosylated digalactosylated structures with bisecting GlcNAc in total IgG glycans*	*GP24 / SUM(GP24 + GP15 + GP19) * 100*
	$F^{total}S1/F^{total}S2$	*Ratio of all fucosylated monosialylated and disialylated structures (+/- bisecting GlcNAc) in total IgG glycans*	*SUM(GP16 + GP18 + GP19) / SUM(GP23 + GP24)*
	FS1/FS2	*Ratio of fucosylated monosialylated and disialylated structures (without bisecting GlcNAc) in total IgG glycans*	*SUM(GP16 + GP18) / GP23*
	FBS1/FBS2	*Ratio of fucosylated monosialylated and disialylated structures (with bisecting GlcNAc) in total IgG glycans*	*GP19 / GP24*
	FBS^{total}/FS^{total}	*Ratio of all fucosylated sialylated structures with and without bisecting GlcNAc in total IgG glycans*	*SUM(GP19 + GP24) / SUM(GP16 + GP18 + GP23)*
	FBS1/FS1	*Ratio of fucosylated monosialylated structures with and without bisecting GlcNAc in total IgG glycans*	*GP19 / SUM(GP16 + GP18)*
	FBS1/(FS1+FBS1)	*The incidence of bisecting GlcNAc in all fucosylated monosialylated structures in total IgG glycans in total IgG glycans*	*GP19 / SUM(GP16 + GP18 + GP19)*
	FBS2/FS2	*Ratio of fucosylated disialylated structures with and without bisecting GlcNAc in*	*GP24 / GP23*

		total IgG glycans	
	FBS2/(FS2+FBS2)	*The incidence of bisecting GlcNAc in all fucosylated disialylated structures in total IgG glycans*	*GP24 / SUM(GP23 + GP24)*

Neutral IgG glycans	$GP1^n$	*The percentage of FA1 glycan in total neutral IgG glycans* (GP^n)	$GP1 / GP^n * 100$
	$GP2^n$	*The percentage of A2 glycan in total neutral IgG glycans* (GP^n)	$GP2 / GP^n * 100$
	$GP4^n$	*The percentage of FA2 glycan in total neutral IgG glycans* (GP^n)	$GP4 / GP^n * 100$
	$GP5^n$	*The percentage of M5 glycan in total neutral IgG glycans* (GP^n)	$GP5 / GP^n * 100$
	$GP6^n$	*The percentage of FA2B glycan in total neutral IgG glycans* (GP^n)	$GP6 / GP^n * 100$
	$GP7^n$	*The percentage of A2G1 glycan in total neutral IgG glycans* (GP^n)	$GP7 / GP^n * 100$
	$GP8^n$	*The percentage of FA2[6]G1 glycan in total neutral IgG glycans* (GP^n)	$GP8 / GP^n * 100$
	$GP9^n$	*The percentage of FA2[3]G1 glycan in total neutral IgG glycans* (GP^n)	$GP9 / GP^n * 100$
	$GP10^n$	*The percentage of FA2[6]BG1 glycan in total neutral IgG glycans* (GP^n)	$GP10 / GP^n * 100$
	$GP11^n$	*The percentage of FA2[3]BG1 glycan in total neutral IgG glycans* (GP^n)	$GP11 / GP^n * 100$
	$GP12^n$	*The percentage of A2G2 glycan in total neutral IgG glycans* (GP^n)	$GP12 / GP^n * 100$
	$GP13^n$	*The percentage of A2BG2 glycan in total neutral IgG glycans* (GP^n)	$GP13 / GP^n * 100$
	$GP14^n$	*The percentage of FA2G2 glycan in total neutral IgG glycans* (GP^n)	$GP14 / GP^n * 100$
	$GP15^n$	*The percentage of FA2BG2 glycan in total neutral IgG glycans* (GP^n)	$GP15 / GP^n * 100$

Neutral IgG glycans -	$G0^n$	*The percentage of agalactosylated structures in total neutral IgG glycans*	$SUM(GP1^n : GP4^n + GP6^n)$
	$G1^n$	*The percentage of monogalactosylated structures in total neutral IgG glycans*	$SUM(GP7^n : GP11^n)$

derived parameters	$G2^n$	*The percentage of digalactosylated structures in total neutral IgG glycans*	*SUM(GP12^n: GP15^n)*
	$F^{n\ total}$	*The percentage of all fucosylated structures (+/- bisecting GlcNAc) in total neutral IgG glycans*	*SUM(GP1^n+ GP4^n+ GP6^n+ GP8^n+ GP9^n+ GP10^n+ GP11^n+ GP14^n+ GP15^n)*
	$FG0^{n\ total}/G0^n$	*The percentage of fucosylation of agalactosylated structures in total neutral IgG glycans*	*SUM(GP1^n+ GP4^n+ GP6^n) / G0^n * 100*
	$FG1^{n\ total}/G1^n$	*The percentage of fucosylation of monogalactosylated structures in total neutral IgG glycans*	*SUM(GP8^n+ GP9^n+ GP10^n+ GP11^n) / G1^n * 100*
	$FG2^{n\ total}/G2^n$	*The percentage of fucosylation of digalactosylated structures in total neutral IgG glycans*	*SUM(GP14^n+ GP15) / G2^n * 100*
	F^n	*The percentage of fucosylated structures (without bisecting GlcNAc) in total neutral IgG glycans*	*SUM(GP1^n+ GP4^n+ GP8^n+ GP9^n+ GP14^n)*
	$FG0^n/G0^n$	*The percentage of fucosylation of agalactosylated structures (without bisecting GlcNAc) in total neutral IgG glycans*	*SUM(GP1^n+ GP4^n) / G0^n * 100*
	$FG1^n/G1^n$	*The percentage of fucosylation of monogalactosylated structures (without bisecting GlcNAc) in total neutral IgG glycans*	*SUM(GP8^n+ GP9^n) / G1^n * 100*
	$FG2^n/G2^n$	*The percentage of fucosylation of digalactosylated structures (without bisecting GlcNAc) in total neutral IgG glycans*	*GP14^n/ G2^n * 100*
	FB^n	*The percentage of fucosylated structures (with bisecting GlcNAc) in total neutral IgG glycans*	*SUM(GP6^n + GP10^n + GP11^n + GP15^n)*
	$FBG0^n/G0^n$	*The percentage of fucosylation of agalactosylated structures (with bisecting GlcNAc) in total neutral IgG glycans*	*GP6^n/ G0^n * 100*
	$FBG1^n/G1^n$	*The percentage of fucosylation of monogalactosylated structures (with bisecting GlcNAc) in total neutral IgG glycans*	*SUM(GP10^n + GP11^n) / G1^n * 100*
	$FBG2^n/G2^n$	*The percentage of fucosylation of digalactosylated structures (with bisecting GlcNAc) in total neutral IgG glycans*	*GP15^n / G2^n * 100*
	FB^n/F^n	*Ratio of fucosylated structures with and without bisecting GlcNAc in total neutral IgG glycans*	*FB^n/ F^n * 100*
	$FB^n/F^{n\ total}$	*The incidence of bisecting GlcNAc in all fucosylated structures in total neutral IgG glycans*	*FB^n/ F$^{n\ total}$ * 100*

Supplementary Table S2 – associations eGFR percentage change per year

Associations between 58 IgG glycans and eGFR change per year. Glycan values are centered and scaled. Shown are the regression coefficient beta and p-value per association. The p-values that remained significant after correction for multiple comparisons (Bonferonni) are highlighted in green. Red represents a positive association and blue a negative association. The basic model was adjusted for age and sex and their interaction. Additionally, the full model was adjusted for smoking, MAP, BMI, Hba1c, duration of type 2 diabetes, nonHDL and HDL.

Basic model			Full model		
Number of tests	58		Number of tests	58	
FDR (Q)	0,05		FDR (Q)	0,05	
FDR α	1,01E-02		FDR α	6,18E-03	
**Glycan trait**	**Beta**	**P-value**	**Glycan trait**	**Beta**	**P-value**
GP6	-1,38	7,76E-04	FBS2/FS2	-0,78	3,20E-03
FBS2/FS2	-1,38	4,16E-04	FBn/Fn	-0,78	2,20E-03
G0n	-1,32	1,84E-03	GP6	-0,76	6,18E-03
FBG2n/G2n	-1,27	1,42E-03	FBG2n/G2n	-0,75	4,11E-03
FBS2/(FS2+FBS2)	-1,21	2,25E-03	FBS2/(FS2+FBS2)	-0,74	5,89E-03
FBStotal/FStotal	-1,16	3,94E-03	FG2S1/(FG2+FG2S1+FG2S2)	-0,74	2,66E-03
GP4	-1,13	6,17E-03	FB/Fntotal	-0,74	3,89E-03
FBS1/(FS1+FBS1)	-0,90	2,34E-02	FBn	-0,72	4,73E-03
FBS1/FS1	-0,90	2,36E-02	FBG1n/G1n	-0,70	5,24E-03
FBn/Fn	-0,77	4,35E-02	FG1S1/(FG1+FG1S1)	-0,60	1,57E-02
FBG1n/G1n	-0,77	4,10E-02	FBG0n/G0n	-0,49	4,77E-02
FB/Fntotal	-0,74	5,20E-02	GP11	-0,47	5,73E-02
FBn	-0,73	5,64E-02	GP2	-0,47	6,96E-02
GP2	-0,64	1,01E-01	GP10	-0,45	6,55E-02
FBG2S2/(FBG2+FBG2S1+FBG2S2)	-0,39	3,04E-01	G0n	-0,39	1,63E-01
FBG0n/G0n	-0,35	3,46E-01	FGS/(FG+FGS)	-0,36	1,56E-01
GP11	-0,30	4,22E-01	GP5	-0,34	1,53E-01
GP3	-0,30	4,31E-01	FS1/FS2	-0,33	1,92E-01
FG2S1/(FG2+FG2S1+FG2S2)	-0,27	4,73E-01	FBStotal/FStotal	-0,31	2,31E-01
GP10	-0,17	6,42E-01	FBG2S2/(FBG2+FBG2S1+FBG2S2)	-0,25	3,06E-01
GP1	-0,14	7,15E-01	GP24	-0,24	3,18E-01
FBG2S1/(FBG2+FBG2S1+FBG2S2)	-0,11	7,71E-01	GP16	-0,23	3,45E-01
GP7	-0,08	8,25E-01	GP13	-0,21	3,95E-01

FG1S1/(FG1+FG1S1)	-0,06	8,74E-01	FBS1/FS1	-0,18	4,86E-01
FS1/FS2	-0,04	9,18E-01	FBS1/(FS1+FBS1)	-0,17	5,26E-01
GP24	0,00	9,89E-01	GP4	-0,13	6,32E-01
FG2S2/(FG2+FG2S1+FG2S2)	0,01	9,71E-01	GP7	-0,11	6,50E-01
GP17	0,10	7,82E-01	FGS/(F+FG+FGS)	-0,09	7,40E-01
FBGS/(FBG+FBGS)	0,15	6,98E-01	GP19	-0,09	7,26E-01
GP19	0,16	6,77E-01	GP15	-0,09	7,38E-01
Fntotal	0,17	6,52E-01	GP20	-0,03	9,01E-01
FG0ntotal/G0n	0,18	6,29E-01	GP21	-0,01	9,74E-01
GP22	0,24	5,18E-01	GP3	0,03	8,98E-01
GP12	0,25	5,02E-01	FG2ntotal/G2n	0,03	8,87E-01
FG1ntotal/G1n	0,26	4,79E-01	GP18	0,07	8,02E-01
FBS1/FBS2	0,30	4,18E-01	FtotalS1/FtotalS2	0,09	7,29E-01
FG2ntotal/G2n	0,33	3,77E-01	GP22	0,11	6,71E-01
GP15	0,34	3,92E-01	GP1	0,11	6,55E-01
FG0n/G0n	0,36	3,33E-01	FBG2S1/(FBG2+FBG2S1+FBG2S2)	0,13	6,19E-01
FGS/(FG+FGS)	0,36	3,50E-01	FG2S2/(FG2+FG2S1+FG2S2)	0,13	5,97E-01
GP13	0,36	3,32E-01	GP17	0,13	5,72E-01
GP21	0,40	2,94E-01	FG1ntotal/G1n	0,14	5,62E-01
GP20	0,41	2,75E-01	FBGS/(FBG+FBGS)	0,16	5,32E-01
GP5	0,55	1,35E-01	Fntotal	0,17	4,85E-01
FBGS/(FB+FBG+FBGS)	0,56	1,41E-01	GP12	0,20	4,03E-01
FtotalS1/FtotalS2	0,65	9,57E-02	FBGS/(FB+FBG+FBGS)	0,26	3,15E-01
GP16	0,69	6,57E-02	FG0ntotal/G0n	0,27	2,82E-01
Fn	0,71	6,37E-02	GP23	0,32	2,29E-01
GP23	0,78	4,81E-02	G1n	0,34	1,98E-01
FG1n/G1n	0,80	3,50E-02	FBS1/FBS2	0,35	1,42E-01
FGS/(F+FG+FGS)	0,85	4,00E-02	G2n	0,36	2,05E-01
GP18	0,89	3,67E-02	GP9	0,44	8,31E-02
G2n	0,98	2,33E-02	GP14	0,49	9,15E-02
GP9	1,05	5,65E-03	FG2n/G2n	0,51	4,86E-02
FG2n/G2n	1,08	6,09E-03	FG0n/G0n	0,51	3,95E-02
GP14	1,12	1,01E-02	GP8	0,61	1,56E-02
GP8	1,18	2,28E-03	Fn	0,70	5,70E-03

G1n	1,43	3,51E-04	FG1n/G1n	0,71	4,71E-03

Supplementary Table S3 – associations eGFR percentage change during total follow-up

Associations between 58 IgG glycans and eGFR change during total follow-up. Glycan values are centered and scaled. Shown are the regression coefficient beta and p-value per association. The p-values that remained significant after correction for multiple comparisons (Bonferonni) are highlighted in green. Red represents a negative association and blue a positive association. The basic model was adjusted for age and sex and their interaction. Additionally, the full model was adjusted for smoking, MAP, BMI, Hba1c, duration of type 2 diabetes, nonHDL, HDL and duration of follow-up.

Percentage change of eGFR during total follow-up					
Basic model			Full model		
Number of tests	58		Number of tests	58	
FDR (Q)	0,05		FDR (Q)	0,05	
FDR α	5,20E-03		FDR α	-	
**Glycan trait**	**Beta**	**P-value**	**Glycan trait**	**Beta**	**P-value**
G0n	-2,39	4,35E-04	G0n	-2,35	1,81E-03
GP6	-2,36	3,53E-04	GP6	-1,92	9,57E-03
GP4	-1,85	5,20E-03	GP4	-1,87	1,03E-02
FG2S1/(FG2+FG2S1+FG2S2)	-1,46	1,52E-02	FBG2S2/(FBG2+FBG2S1+FBG2S2)	-1,81	5,22E-03
FBG2S2/(FBG2+FBG2S1+FBG2S2)	-1,35	2,64E-02	FG2S1/(FG2+FG2S1+FG2S2)	-1,74	7,74E-03
FBG2n/G2n	-1,34	3,58E-02	FG2S2/(FG2+FG2S1+FG2S2)	-1,24	6,55E-02
FBn	-1,23	4,42E-02	FG1S1/(FG1+FG1S1)	-1,22	6,27E-02
FB/Fntotal	-1,20	4,91E-02	GP21	-1,11	1,05E-01
FBn/Fn	-1,20	5,01E-02	FBG2n/G2n	-0,98	1,58E-01
FBG1n/G1n	-1,12	6,39E-02	FGS/(FG+FGS)	-0,81	2,31E-01
FG1S1/(FG1+FG1S1)	-1,10	6,70E-02	FBS2/(FS2+FBS2)	-0,79	2,71E-01
FBS2/(FS2+FBS2)	-0,98	1,25E-01	FBn	-0,71	2,92E-01
FBS2/FS2	-0,92	1,44E-01	FBS2/FS2	-0,70	3,16E-01
FG2S2/(FG2+FG2S1+FG2S2)	-0,67	2,75E-01	FBStotal/FStotal	-0,70	3,14E-01
GP2	-0,65	2,97E-01	FBn/Fn	-0,68	3,14E-01

FBStotal/FStotal	-0,61	3,42E-01	FB/Fntotal	-0,68	3,15E-01
FBG0n/G0n	-0,58	3,30E-01	FBG1n/G1n	-0,61	3,60E-01
GP21	-0,50	4,09E-01	GP2	-0,47	4,94E-01
FGS/(FG+FGS)	-0,49	4,34E-01	GP5	-0,46	4,66E-01
GP5	-0,40	5,03E-01	FBGS/(FBG+FBGS)	-0,44	5,08E-01
FBS1/(FS1+FBS1)	-0,26	6,90E-01	GP24	-0,43	5,09E-01
GP10	-0,24	6,87E-01	GP22	-0,42	5,28E-01
FBS1/FS1	-0,22	7,32E-01	GP1	-0,40	5,47E-01
GP3	-0,14	8,21E-01	FBS1/(FS1+FBS1)	-0,34	6,22E-01
GP11	-0,14	8,20E-01	FBS1/FS1	-0,33	6,35E-01
FG0ntotal/G0n	-0,14	8,21E-01	FG0ntotal/G0n	-0,24	7,17E-01
Fntotal	-0,12	8,41E-01	Fntotal	-0,23	7,26E-01
GP20	-0,10	8,70E-01	FBG0n/G0n	-0,20	7,64E-01
GP1	-0,07	9,06E-01	FG1ntotal/G1n	-0,10	8,75E-01
GP22	-0,02	9,70E-01	GP16	-0,06	9,25E-01
GP24	0,03	9,66E-01	FBG2S1/(FBG2+FBG2S1+FBG2S2)	-0,02	9,81E-01
FG1ntotal/G1n	0,07	9,01E-01	FG2ntotal/G2n	-0,01	9,91E-01
FG2ntotal/G2n	0,09	8,81E-01	GP3	0,00	9,95E-01
GP16	0,17	7,77E-01	GP23	0,04	9,56E-01
GP7	0,21	7,20E-01	FG0n/G0n	0,10	8,86E-01
GP17	0,23	6,96E-01	GP11	0,15	8,23E-01
FBGS/(FBG+FBGS)	0,26	6,65E-01	GP17	0,23	7,16E-01
FBG2S1/(FBG2+FBG2S1+FBG2S2)	0,27	6,58E-01	GP10	0,26	6,88E-01
FS1/FS2	0,39	5,17E-01	GP7	0,32	6,23E-01
FG0n/G0n	0,46	4,41E-01	GP20	0,38	5,57E-01
GP23	0,65	3,05E-01	FBGS/(FB+FBG+FBGS)	0,47	4,84E-01
GP12	0,79	1,89E-01	GP19	0,54	4,09E-01
FGS/(F+FG+FGS)	0,86	1,95E-01	Fn	0,56	4,12E-01

GP13	0,87	1,47E-01	FGS/(F+FG+FGS)	0,56	4,37E-01
GP19	0,91	1,32E-01	FG1n/G1n	0,58	3,85E-01
FG2n/G2n	0,93	1,41E-01	FG2n/G2n	0,63	3,62E-01
Fn	1,06	8,44E-02	GP13	0,77	2,29E-01
FG1n/G1n	1,11	6,76E-02	GP12	0,82	2,06E-01
FtotalS1/FtotalS2	1,16	6,35E-02	FS1/FS2	0,86	1,97E-01
GP15	1,16	6,78E-02	GP18	0,90	2,32E-01
FBGS/(FB+FBG+FBGS)	1,17	5,55E-02	GP15	1,39	4,23E-02
GP18	1,20	8,11E-02	FtotalS1/FtotalS2	1,53	2,19E-02
FBS1/FBS2	1,43	1,63E-02	GP9	1,59	1,79E-02
GP9	1,72	4,52E-03	FBS1/FBS2	1,67	9,02E-03
G2n	1,98	4,30E-03	G2n	1,88	1,37E-02
GP8	1,99	1,26E-03	GP8	1,89	5,07E-03
GP14	2,12	2,38E-03	GP14	2,04	7,83E-03
G1n	2,36	2,33E-04	G1n	2,37	8,82E-04

Supplementary Table S4 – associations ACR percentage change per year

Associations between 58 IgG glycans and ACR change per year. Glycan values are centered and scaled. Shown are the regression coefficient beta and p-value per association. The p-values that remained significant after correction for multiple comparisons (Bonferonni) are highlighted in green. Red represents a positive association and blue a negative association. The basic model was adjusted for age and sex and their interaction. Additionally, the full model was adjusted for smoking, MAP, BMI, Hba1c, duration of type 2 diabetes, ACE-inhibitor, nonHDL and HDL.

ACR percentage change per year					
Basic model			Full model		
Number of tests	58		Number of tests	58	
FDR (Q)	0,05		FDR (Q)	0,05	
FDR α	-		FDR α	-	
**Glycan trait**	**Beta**	**P-value**	**Glycan trait**	**Beta**	**P-value**
FBGS/(FBG+FBGS)	-35,40	4,69E-02	FBGS/(FBG+FBGS)	-30,47	1,44E-01
FG1n/G1n	-33,71	6,37E-02	GP3	-29,43	1,91E-01
FBG2S1/(FBG2+FBG2S1+FBG2S2)	-30,80	9,55E-02	FBG2S1/(FBG2+FBG2S1+FBG2S2)	-29,04	1,81E-01
Fn	-26,57	1,48E-01	GP1	-27,09	2,02E-01
GP3	-26,20	1,71E-01	FBGS/(FB+FBG+FBGS)	-21,53	3,16E-01
FBGS/(FB+FBG+FBGS)	-26,05	1,51E-01	FG1n/G1n	-20,92	3,31E-01
FG0n/G0n	-23,41	1,92E-01	GP19	-19,98	3,47E-01
GP4	-22,70	2,62E-01	FBS1/(FS1+FBS1)	-19,94	3,69E-01
FG2S2/(FG2+FG2S1+FG2S2)	-22,22	2,20E-01	FBS1/FS1	-18,81	3,94E-01
GP9	-22,07	2,28E-01	GP17	-17,86	4,14E-01
GP1	-21,66	2,37E-01	GP13	-15,66	4,51E-01
G0n	-19,90	3,39E-01	GP7	-15,59	4,55E-01

FBG2S2/(FBG2+FBG2S1+FBG2S2)	-18,88	2,93E-01	FBStotal/FStotal	-15,04	4,95E-01
FG2n/G2n	-18,48	3,40E-01	GP12	-14,80	4,78E-01
FBS1/(FS1+FBS1)	-15,66	4,16E-01	GP21	-14,61	5,13E-01
FBS1/FS1	-14,84	4,38E-01	FBS1/FBS2	-14,26	4,88E-01
GP19	-12,74	4,81E-01	GP2	-14,12	5,21E-01
GP23	-12,24	5,17E-01	GP20	-13,55	5,07E-01
FBStotal/FStotal	-11,57	5,48E-01	G0n	-12,84	6,02E-01
GP20	-8,90	6,18E-01	GP4	-12,76	5,94E-01
GP2	-7,57	6,80E-01	Fn	-11,73	5,91E-01
GP24	-7,53	6,67E-01	FG2n/G2n	-9,12	6,88E-01
GP21	-6,24	7,29E-01	FG2S2/(FG2+FG2S1+FG2S2)	-8,96	6,77E-01
FBS1/FBS2	-4,97	7,80E-01	GP24	-8,42	6,78E-01
GP17	-3,29	8,51E-01	FBG2S2/(FBG2+FBG2S1+FBG2S2)	-7,27	7,22E-01
GP7	-3,21	8,57E-01	GP9	-6,00	7,83E-01
FG2ntotal/G2n	-0,80	9,65E-01	GP14	-3,42	8,91E-01
GP22	2,03	9,09E-01	FG0n/G0n	-3,19	8,81E-01
GP13	2,77	8,78E-01	GP23	-2,13	9,24E-01
Fntotal	3,05	8,64E-01	G2n	-1,28	9,59E-01
FG0ntotal/G0n	4,60	7,97E-01	FBS2/(FS2+FBS2)	-1,10	9,62E-01
GP12	4,90	7,85E-01	GP5	-0,54	9,79E-01
FG1S1/(FG1+FG1S1)	4,99	7,80E-01	GP22	0,05	9,98E-01
FGS/(FG+FGS)	5,20	7,79E-01	FBS2/FS2	0,24	9,91E-01
GP16	5,76	7,50E-01	GP6	3,03	8,99E-01

FG1ntotal/G1n	6,02	7,37E-01	FGS/(FG+FGS)	8,74	6,84E-01
FBS2/(FS2+FBS2)	8,92	6,39E-01	FBG0n/G0n	9,03	6,70E-01
FBS2/FS2	9,15	6,23E-01	GP11	9,19	6,58E-01
GP6	10,39	6,04E-01	FS1/FS2	10,63	6,18E-01
FG2S1/(FG2+FG2S1+FG2S2)	12,04	5,01E-01	FG2ntotal/G2n	12,58	5,59E-01
GP14	12,65	5,53E-01	GP18	12,75	5,98E-01
GP11	12,75	4,79E-01	FtotalS1/FtotalS2	13,29	5,30E-01
GP5	13,43	4,63E-01	GP15	13,42	5,45E-01
GP8	13,94	4,57E-01	FG0ntotal/G0n	14,52	4,93E-01
G2n	15,49	4,63E-01	FGS/(F+FG+FGS)	14,65	5,29E-01
FGS/(F+FG+FGS)	15,52	4,40E-01	GP8	14,75	4,96E-01
GP18	15,92	4,46E-01	FG1S1/(FG1+FG1S1)	16,34	4,36E-01
G1n	20,64	2,95E-01	Fntotal	17,32	4,09E-01
FS1/FS2	21,33	2,34E-01	FBn/Fn	17,34	4,24E-01
FtotalS1/FtotalS2	21,95	2,37E-01	FG1ntotal/G1n	18,20	3,84E-01
FBG2n/G2n	26,96	1,62E-01	FB/Fntotal	18,29	4,01E-01
FBG0n/G0n	28,57	1,09E-01	FBn	19,94	3,59E-01
FBn/Fn	29,34	1,09E-01	FG2S1/(FG2+FG2S1+FG2S2)	22,62	2,75E-01
FB/Fntotal	30,18	9,98E-02	GP16	23,75	2,56E-01
GP15	30,68	1,06E-01	FBG1n/G1n	24,36	2,54E-01
FBn	30,82	9,23E-02	G1n	25,99	2,63E-01
FBG1n/G1n	35,30	5,09E-02	FBG2n/G2n	28,22	2,04E-01
GP10	40,03	2,56E-02	GP10	30,30	1,50E-01

Supplementary Table S5 – associations percentage change of ACR during total follow-up

Associations between 58 IgG glycans and ACR change during total follow-up. Glycan values are centered and scaled. Shown are the regression coefficient beta and p-value per association. The p-values that remained significant after correction for multiple comparisons (Bonferonni) are highlighted in green. Red represents a positive association and blue a negative association. The basic model was adjusted for age and sex and their interaction. Additionally, the full model was adjusted for smoking, MAP, BMI, Hba1c, duration of type 2 diabetes, ACE-inhibitor, nonHDL, HDL and duration of follow-up.

Percentage change of ACR during total follow-up					
Basic model			Full model		
Number of tests	58		Number of tests	58	
FDR (Q)	0,05		FDR (Q)	0,05	
FDR α	-		FDR α	-	
**Glycan trait**	**Beta**	**P-value**	**Glycan trait**	**Beta**	**P-value**
FBGS/(FBG+FBGS)	-196,80	3,58E-02	FBGS/(FBG+FBGS)	-173,92	1,16E-01
FG1n/G1n	-159,70	9,53E-02	GP1	-166,81	1,41E-01
FBG2S1/(FBG2+FBG2S1+FBG2S2)	-148,91	1,25E-01	FBG2S1/(FBG2+FBG2S1+FBG2S2)	-164,98	1,53E-01
FBGS/(FB+FBG+FBGS)	-141,28	1,39E-01	FBS1/(FS1+FBS1)	-141,83	2,32E-01
FG2S2/(FG2+FG2S1+FG2S2)	-133,49	1,61E-01	FBS1/FS1	-133,03	2,59E-01
Fn	-124,04	1,99E-01	FG1n/G1n	-126,29	2,70E-01
FBG2S2/(FBG2+FBG2S1+FBG2S2)	-123,58	1,89E-01	GP3	-121,85	2,98E-01
GP4	-122,42	2,49E-01	FBGS/(FB+FBG+FBGS)	-119,86	2,93E-01
FBS1/(FS1+FBS1)	-116,92	2,48E-01	FBStotal/FStotal	-117,82	3,17E-01
FG0n/G0n	-113,70	2,27E-01	GP4	-111,86	3,80E-01
FBS1/FS1	-113,01	2,61E-01	G0n	-103,71	4,29E-01
G0n	-112,07	3,05E-01	GP19	-101,72	3,68E-01
GP1	-110,59	2,53E-01	Fn	-91,40	4,30E-01
FBStotal/FStotal	-105,84	2,96E-01	FG2S2/(FG2+FG2S1+FG2S2)	-77,22	4,98E-01
GP3	-83,79	3,98E-01	GP7	-70,40	5,24E-01

GP19	-82,62	3,85E-01	FG0n/G0n	-67,04	5,54E-01
GP23	-75,28	4,48E-01	FBG2S2/(FBG2+FBG2S1+FBG2S2)	-65,52	5,43E-01
GP24	-62,30	5,01E-01	GP2	-58,26	6,17E-01
GP2	-52,80	5,83E-01	GP17	-57,51	6,19E-01
GP9	-45,69	6,33E-01	GP12	-55,13	6,19E-01
GP7	-42,79	6,47E-01	GP13	-55,07	6,19E-01
FG2n/G2n	-42,02	6,77E-01	FBS1/FBS2	-50,31	6,44E-01
GP17	-37,79	6,81E-01	GP24	-46,95	6,64E-01
GP22	-36,32	6,91E-01	GP5	-40,56	7,14E-01
GP20	-21,00	8,23E-01	FG2n/G2n	-36,13	7,62E-01
GP12	-10,16	9,14E-01	GP21	-29,96	7,98E-01
GP13	-5,97	9,50E-01	GP20	-27,10	8,04E-01
GP21	-5,82	9,51E-01	GP23	-19,84	8,66E-01
FBS1/FBS2	7,30	9,38E-01	GP22	-12,34	9,20E-01
FGS/(FG+FGS)	20,97	8,29E-01	FBS2/(FS2+FBS2)	-8,56	9,44E-01
FG0ntotal/G0n	28,17	7,64E-01	FBS2/FS2	-1,97	9,87E-01
FBS2/(FS2+FBS2)	31,89	7,50E-01	GP9	1,87	9,87E-01
FBS2/FS2	33,98	7,29E-01	GP14	8,39	9,50E-01
GP5	38,65	6,86E-01	G2n	22,05	8,67E-01
FG2ntotal/G2n	38,67	6,80E-01	GP6	24,20	8,49E-01
Fntotal	40,69	6,64E-01	FG0ntotal/G0n	41,68	7,11E-01
GP6	46,19	6,61E-01	FG2ntotal/G2n	57,87	6,04E-01
GP14	55,76	6,19E-01	FGS/(FG+FGS)	63,89	5,74E-01
FG1S1/(FG1+FG1S1)	58,72	5,33E-01	Fntotal	67,70	5,42E-01
FG2S1/(FG2+FG2S1+FG2S2)	59,00	5,31E-01	GP8	72,56	5,31E-01
FG1ntotal/G1n	61,75	5,11E-01	GP15	83,93	4,76E-01
G2n	62,91	5,71E-01	FG1ntotal/G1n	85,81	4,38E-01

GP18	74,27	4,96E-01	GP18	90,39	4,83E-01
GP8	77,01	4,34E-01	FBG0n/G0n	92,85	4,10E-01
FGS/(F+FG+FGS)	80,96	4,41E-01	GP11	98,78	3,72E-01
GP16	87,15	3,58E-01	FGS/(F+FG+FGS)	110,69	3,71E-01
FBG2n/G2n	116,45	2,53E-01	FBn/Fn	119,62	3,00E-01
GP11	117,30	2,17E-01	FB/Fntotal	122,55	2,90E-01
GP15	132,35	1,84E-01	FG2S1/(FG2+FG2S1+FG2S2)	123,81	2,61E-01
FBG0n/G0n	141,73	1,31E-01	FG1S1/(FG1+FG1S1)	124,09	2,67E-01
G1n	143,39	1,65E-01	FS1/FS2	125,97	2,68E-01
FBn/Fn	148,65	1,23E-01	FBG2n/G2n	127,99	2,82E-01
FB/Fntotal	150,25	1,20E-01	FBn	129,85	2,62E-01
FBn	155,57	1,07E-01	FBG1n/G1n	143,63	2,07E-01
FS1/FS2	165,15	8,07E-02	FtotalS1/FtotalS2	143,79	2,01E-01
FBG1n/G1n	173,56	6,89E-02	GP16	171,82	1,23E-01
FtotalS1/FtotalS2	178,71	6,73E-02	G1n	172,22	1,62E-01
GP10	212,45	2,46E-02	GP10	183,49	1,01E-01
